# PGC-1α agonist ZLN005 ameliorates OVA-induced asthma in BALB/c mice through modulating the NF-κB–p65/NLRP3 pathway

**DOI:** 10.22038/ijbms.2025.83166.17982

**Published:** 2025

**Authors:** Rui Fang, Yan Cheng, Ping Chen, Jing Hu, Liqi Yang

**Affiliations:** 1 Department of Pediatrics, The Second Affiliated Hospital of Anhui Medical University, Hefei 230601, Anhui, China

**Keywords:** Asthma, Inflammation, NF-kappa B, NLR family, Pyrin domain containing 3 – protein, Th2 cells

## Abstract

**Objective(s):**

Asthma is a complex inflammatory disease of the lungs marked by increased infiltration of leukocytes into the airways, which restricts respiratory function. Proliferator-activated receptor-γ coactivator-1 alpha (PGC-1α) has been recognized as an essential immunomodulator and has the potential as a novel anti-inflammatory target in asthma. The current study aims to investigate the functions of PGC-1α in ovalbumin (OVA)-sensitized asthmatic mice and underlying mechanisms.

**Materials and Methods:**

BALB/c mouse asthma model was induced by OVA *in vivo*. The therapeutic effects of PGC-1α agonist (ZLN005) on asthma were assessed by histological and biochemical analysis. In addition, we integrated real-time qPCR, western blotting, and immunofluorescence analysis to reveal the underlying mechanism.

**Results:**

In the lung tissue of asthmatic mice, PGC-1α levels were down-regulated. Diff-Quik staining indicated that ZLN005 therapy on asthmatic mice reduced the number of inflammatory cells (eosinophilic granulocytes, neutrophils, lymphocytes, and mononuclear macrophages) in bronchoalveolar lavage fluid (BALF), ameliorated pathologic alterations in lung tissues. ZLN005 alleviated airway structure and inflammation, as well as down-regulating the serum immunoglobulin E (IgE), OVA-specific IgE, and T-helper 2 (Th2) cytokines (interleukin (IL)-4, IL-5, and IL-13) expression. Mechanistically, the results showed that ZLN005, through the NF-κB-p65 axis, prominently inhibited the activation of the NLRP3 inflammasome and reduced the levels of the NLRP3 downstream targets IL-1β and IL-18.

**Conclusion:**

PGC-1α agonist (ZLN005) regulated lung inflammation in asthmatic mice by inhibiting the NF-κB-p65/NLRP3 signaling pathway, supporting that ZLN005 may be a candidate for future asthma treatment.

## Introduction

Asthma is a complicated heterogeneous disease characterized by persistent airway inflammation, wheezing, shortness of breath, chest tightness, and dyspnea caused by airway congestion (1). According to the International Study of Asthma and Allergies in Childhood (ISAAC), 6.9 percent of adolescents worldwide have severe asthma. This ranges from 3.8 percent in the Asia-Pacific region and Northern and Eastern Europe to 11.3 percent in North America (2). The use of inhaled corticosteroids and long-acting beta-2 adrenergic receptor agonists is recommended as first-line treatment in the Global Asthma Initiative guidelines (3). However, there are certain side effects and adverse reactions associated with these drug interventions, so there is an urgent need to look for new therapeutic options.

Exposure to allergens, bacterial infections, impaired antiviral immunity, and viral respiratory infections are common triggers of asthma exacerbations (4). The pathogenesis of asthma involves the activation of allergens by immune cells such as eosinophils, neutrophils, and lymphocytes. This stimulation induces a transition in the immunoglobulin (Ig)E isotype, culminating in pulmonary eosinophilia and bronchoconstriction (5). Moreover, approximately 50 percent of cases of mild to moderate asthma and a large proportion of cases of severe asthma are caused by T helper 2 (Th2)-dependent inflammation (6). Dysregulated Th2 inflammation contributes to the primary pathological mechanism in asthma, driven by Th2 cells that secrete Th2 cytokines (interleukin (IL)-4, IL-5, and IL-13), thereby instigating specific inflammatory cascades and perpetuating an inflammatory response (7). As a result, therapeutic interventions for asthma primarily target inflammation predominantly. Ovalbumin (OVA) is a commonly utilized antigen in experimental animal models of inducible asthma. The combination of OVA with the Al (OH)_3_ adjuvant elicits a typical Th2-type response, contributing to the pathogenesis of asthma (8). Here, our study constructed a model of OVA-sensitized asthma to explore potential therapeutic mechanisms.

Proliferator-activated receptor gamma coactivator 1α (PGC-1α) is a ligand-activated transcription factor that is part of the nuclear hormone receptor superfamily (4). Remarkably, PGC-1α has been recognized as an essential immunomodulator and has the potential as a novel anti-inflammatory target for asthma (9, 10). A previous study has shown that PGC-1α is reduced in the lung tissues of guinea pigs with bronchial asthma models (11). Concurrently, using PGC-1α agonists treating anti-inflammatory agents has been identified as a promising avenue for asthma and pulmonary disease (12). As a PGC-1α agonist, ZLN005 functions by up-regulating PGC-1α activity and exerts a protective effect in alveolar epithelial cell lines (13). There is no documented evidence of the therapeutic efficacy of PGC-1α in an allergic asthma model.

Recent research indicates that the inflammasome, a complex of inflammatory proteins, identifies allergens, facilitating innate immunity (14). Growing clinical evidence has highlighted the key role of NOD-like Receptor Family Pyrin Domain Containing 3 (NLRP3) inflammasome activation in the pathogenesis of asthma (15). Using NLRP3 mutant mice, Ma *et al*. demonstrated that the inhibition of NLRP3 attenuates the inflammatory response and pathogenesis associated with allergic asthma (16).

Nuclear factor κB (NF-κB) is an essential transcription factor in regulating inflammation and immunity** (17).** Asthma is exacerbated by upper respiratory tract infections that trigger NF-κB (18). NF-κB serves a pivotal role in the priming signal that is necessary for NLRP3 inflammasome activation. It initiates the transcriptional expression of NLRP3 and pro-IL-1β in response to a range of cytokines and pattern-recognition receptor ligands (19). Previous research has indicated that pharmacological inhibition of the NF-kB-NLRP3 signaling pathway could attenuate the allergic airway inflammation of asthma (20). Furthermore, PGC-1α regulates the pro-inflammatory cytokines synthesis through physical interaction with the P65 NF-κB subunit (21). However, whether PGC-1a can participate in asthma treatment by regulating the NFκB-NLRP3 signaling pathway has not been investigated. Accordingly, our study explored the ability of PGC-1α agonist (ZLN005) to ameliorate allergic asthma in OVA-sensitized mice via the NFκB-NLRP3 signaling pathway.

## Materials and Methods

### Human study design

Thirty children with allergic asthma and 30 generally age- and sex-matched healthy controls (those without a history of asthma or other allergic disorders) were recruited from Anhui Medical University. The research follows the tenets of the Declaration of Helsinki (1975) and its amendments. Furthermore, informed consent was obtained from the parents of all participants.

### Mice

Postnatal day 5 (P5) BALB/c mice were provided by Jiangsu Huachuang Sino PharmaTech Co., Ltd. During rearing, experiments were performed in a laboratory with a controlled environment animal room (temperature, 21 °C–23 °C; relative humidity, 45%–55%; frequent ventilation) and kept in a SPF environment. All animal experiments complied with ARRIVE guidelines and carried out following Guidance on the operation of the Animals Act 1986 and associated guidelines. In addition, all animal experiments were approved by the Animal Care and Use Committee of Anhui Medical University.

### OVA-sensitized allergic asthma model

OVA-induced asthmatic mice model is frequently utilized to explore the pathogenesis of asthma (22). Moreover, Al (OH)_3_ is associated with exposures of the respiratory mucosa, indicating its potential use as a model adjuvant therapy for airway exposure (23). The OVA-induced allergic asthma model was established according to previous research (24). Four groups of mice were randomly assigned: The control group, the Asthma group, the Asthma+L-ZLN005 (6 mg/kg) group, and the Asthma+H-ZLN005 (12 mg/kg) group. Mice in the model and drug treatment groups were sensitized on postnatal d 5 (P5) and P10 by intraperitoneal injection of 20 μl normal saline containing 10 μg OVA (Macklin, A800616) and 1mg Al (OH)_3 _(Sangon Biotech, A510023). Next, the mice were stimulated thrice with 3% OVA atomized solution for 10 min at P18, P19, and P20. The control group received PBS as stimulation. To evaluate the effect of PGC-1α on asthma, we administered 6/12 mg/kg PGC-1α agonist (ZLN005) by intraperitoneal injection on P18, P19, and P20. The asthma group was treated with an equal solvent. The BALB/c mice were sacrificed 24 hr after stimulation with OVA on P21. In the meantime, the blood, lung tissues, and alveolar lavage fluid were collected for subsequent experiments.

### Collection of bronchoalveolar lavage fluid (BALF) and Diff-Quik staining

To collect BALF, 500 μl of ice-cold PBS was infused 3 times into the lungs. The infusion was stopped with the tracheal cannula. Gently rubbed the mouse’s chest for 15-20 sec to absorb the BALF (25). Total inflammatory cells in BALF were determined by cell counting using a hemocytometer (Shanghai Qiujing Biology, 02270113). Inflammatory cells were identified by applying a Diff-Quik stain to BAL fluid. First, BAL fluid samples were smeared on a glass slide and dried. Cells from 10 ul of lavage were smeared, and rapid staining was performed with Diff-Quik (Leagene Biotechnology, DA0098). Under microscopic examination (OLYMPUS, DP73), the percentages of mononuclear macrophages, lymphocytes, eosinophilic granulocytes, inflammatory cells, and neutrophils were documented (26).

### Quantitative real-time PCR

Total RNA was extracted from lung tissues using the TRIpure reagent (BioTeke, RP1001). cDNA was synthesized using an All-in-One first strand supermix kit (Magen Biotech, MD80101). To quantify mRNA expression, cDNAs were amplified by RT-qPCR using the SYBR Green kit (Solarbio, SR4110). GAPDH was used as an internal reference for mRNA to calculate the relative transcription level of the target gene using the 2^−ΔΔCt^ technique. Quantitative fluorescence analysis was performed using an ExicyclerTM 96 fluorescence quantifier manufactured by BIONEER **(27)**. The primer sequences used for all genes were as follows:

PGC-1α, forward primer: 

5’-GAACAAGACTATTGAGCGAACC-3’,

PGC-1α, reverse primer: 5’-GAGTGGCTGCCTTGGGTA-3’;

IL-1β, forward primer: 5’-TTCCCATTAGACAACTGC-3’,

IL-1β, reverse primer: 5’-GATTCTTTCCTTTGAGGC-3’;

IL-18, forward primer: 5’-CACGCTTTACTTTATACCT-3’,

IL-18, reverse primer: 5’-CACAGCCAGTCCTCTTA-3’.

### Western blot analysis

The protein samples were extracted using RIPA buffer (Proteintech, PR20001) containing protease inhibitors PMSF (Proteintech, PR20032) and quantified through a BCA protein assay kit (Proteintech, PK10026). The extracted proteins were subjected to the SDS-PAGE and then transferred to the PVDF membranes (Thermo Fisher Scientific, LC2005). After blocking, the membranes were incubated with the primary antibodies against PGC-1α (ABclonal, A12348, 1:1000), NLRP3 (ABclonal, A21906, 1:3000), and p65 (ABclonal, A2547, 1:1000) overnight at 4℃. As a loading control, the data were normalized using the antibodies β-actin (Proteintech, 66009-1-Ig, 1:20000) and Histone H3 (Proteintech, 17168-1-AP, 1:2000). Representative protein images were visualized using the ECL kit (Proteintech, PK10003) **(**28**)**.

### Enzyme-linked immunosorbent assay (ELISA)

Whole blood samples were centrifuged at 3500 rpm for 10 min to separate the serum, which was stored at -80 °C until use. After thawing, the samples were centrifuged again, and the biochemical indicators, including PGC-1α (Wuhan Fine Biotech, EM0534), IgE (Wuhan Fine Biotech, EM0211), and OVA-sIgE (Wuhan Fine Biotech, EM1254) were quantified using the respective detection kit.

The lung tissues were homogenized in normal saline and centrifuged at 2500 rpm for 10 min, and the supernatant was isolated for further evaluation. IL-4 (MultiSciences Biotech, EK204), IL-5 (MultiSciences Biotech, EK205), and IL-13 (MultiSciences Biotech, EK213) from BALF and lung tissues were analyzed according to the manufacturer’s instructions. In all experiments, absorbance was determined using BIOTEK’s enzyme-labeled instrument (ELX-800). A standard curve produced on each plate served as the basis for determining the concentrations (29).

### Histological analysis

Lung tissues were collected and fixed in 4% paraformaldehyde (Macklin, P804536) for 24 hr. After dehydration through graded ethanol, lung tissues were embedded in paraffin. After deparaffinization and dehydration, sections (5 μm) were cut and stained with hematoxylin and eosin (H&E, Solarbio, H8070) and periodic acid-Schiff (PAS, Leagene Biotechnology, DG0005) as described by Wang *et al*.** (30)**. Images were captured using an upright fluorescence microscope (Olympus, BX53) at 200× magnification to scan the entire section. Lung injury was scored for alveolar and interstitial inflammation and interstitial hemorrhage.

### Immunofluorescence staining

The specimens were deparaffinized in xylene, rehydrated through graded alcohols, and rinsed in PBS. After antigen retrieval, the specimens were blocked with bovine serum albumin (BSA, Sangon Biotech, A602440-0050) and then incubated with primary antibodies NLRP3 (ABclonal, A21906) and p-p65^ser536^ (Affinity, AF2006) at 4 ℃ overnight and then incubated with secondary antibody Cy3-Goat Anti-Rabbit IgG (Proteintech, SA00009-2) at room temperature for 60 min. The nuclei were treated with 4’,6- Diamidino-2-phenylindole Dihydrochloride (DAPI, Aladdin, D106471-5mg). Finally, the specimens were mounted with neutral gum and imaged under a microscope (OLYMPUS, BX53).

### Statistical analysis

All data were presented as mean ± standard deviation (SD). Statistical analysis was performed using the GraphPad Prism 8 software. One-way analysis of variance followed by multiple comparisons was used to compare differences between groups. A difference that was deemed statistically significant was defined as having a value of *P*<0.05.

## Results

### PGC-1α is down-regulated in asthma patients and OVA-sensitized asthma mice, alleviates lung injury in asthma mice

**Figure 1:**
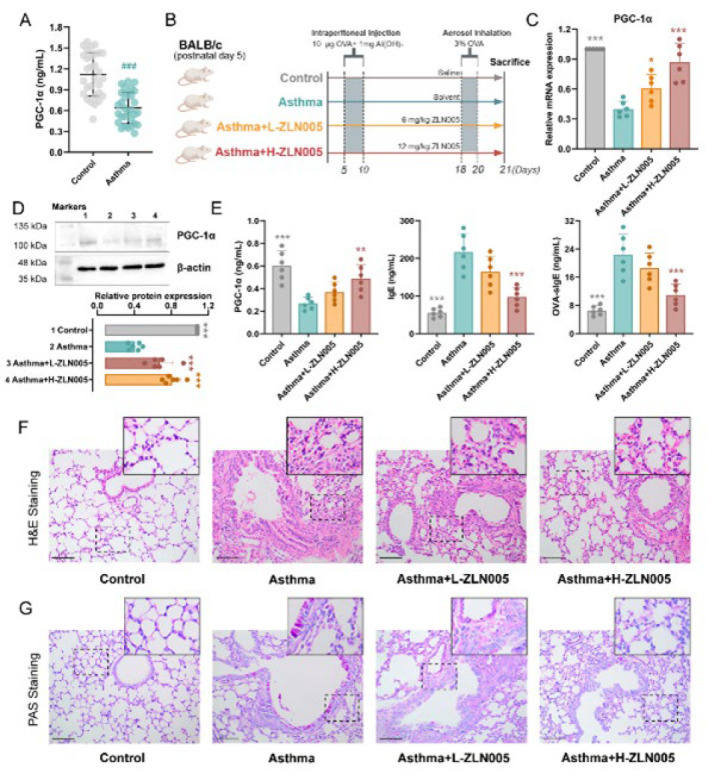
PGC-1α is down-regulated in asthma patients and OVA-induced asthmatic mice and alleviates lung injury in asthmatic mice

To investigate whether PGC-1α is involved in asthma, we first examined its expression in lung tissues of asthmatic patients and mice. ELISA demonstrated that the expression level of PGC-1α was reduced in lung tissues of asthmatic patients (Figure 1A). The procedure of the animal experiment is shown in Figure 1B. Real-time qPCR, western blot, and ELISA indicated that the content of PGC-1α is reduced in asthmatic mice, while the treatment of PGC-1α agonist (ZLN005) alleviated the down-regulation of PGC-1α (Figure 1C-1E). The serum IgE and OVA-specific IgE levels were markedly increased in OVA-sensitized asthma mice, and this tendency was decreased in PGC-1α agonist-treated mice (Figure 1E).

Inflammation of the airways is considered a central pathological feature of asthma. HE and PAS staining were used to observe the histological morphology and inflammatory cell infiltration of the lung tissue sections in the mouse model of severe asthma. HE staining showed a partially disappeared alveolar structure and severe intercellular edema with tissue congestion in asthma mice. Upon administration with ZLN005, lung damage was considerably reduced (Figure 1F). PAS staining showed that the goblet cells reacted positively, suggesting cell hyperplasia in asthma mice. The pathological symptoms in asthma mice were alleviated by ZLN005 therapy (Figure 1G).

### ZLN005 reduces the accumulation of inflammatory cells in BALF

**Figure 2:**
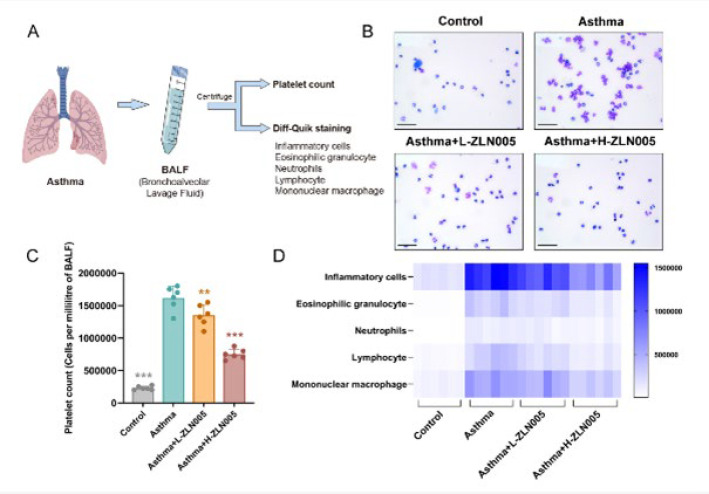
ZLN005 reduced the accumulation of inflammatory cells in BALF

Diff-Quik staining was performed to determine the percentage of inflammatory cells in the BALF (Figure 2B). Mice in the OVA group had a much higher percentage of inflammatory cells than those in the ZLN005 group. Moreover, the total number of inflammatory cells was decreased significantly in ZLN005 administered groups compared with that in the asthma group (Figure 2C). The number of mononuclear macrophages, lymphocytes, eosinophilic granulocytes, and neutrophils were markedly up-regulated in asthmatic mice. At the same time, ZLN005 alleviated this tendency (Figure 2D). The results above indicated the protective attributes of ZLN005 in asthma.

### ZLN005 mitigates Th2 cells inflammatory restimulation in OVA-induced asthmatic mice

**Figure 3:**
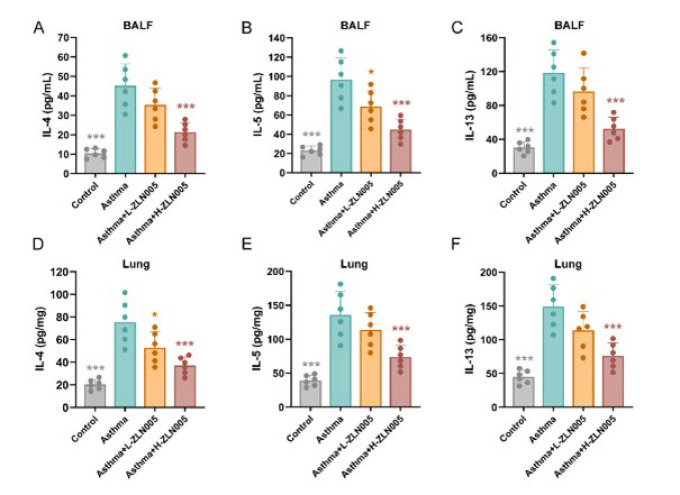
ZLN005 mitigated Th2 cells inflammatory restimulation in OVA-induced asthmatic mice

IL-4, IL-5, and IL-13 are mediators driven by Th2 cells and involved in respiratory inflammation (6). To further determine the inhibitory effects of ZLN005 on cytokine levels in OVA-induced mice, IL-4, IL-5, and IL-13 levels in the BALF and lung tissues were measured using ELISA. Compared with control mice, IL-4, IL-5, and IL-13 concentrations were significantly increased in mice with asthma. Mice in ZLN005 treatment groups had marked suppression of IL-4, IL-5, and IL-13 levels compared with those in the OVA group (Figure 3A–F). Collectively, these findings demonstrated that ZLN005 inhibited type 2 inflammation and ameliorated OVA-induced asthmatic mice.

### ZLN005 suppresses the activation of NF-κB–p65/NLRP3 pathway in OVA-induced asthmatic mice

**Figure 4:**
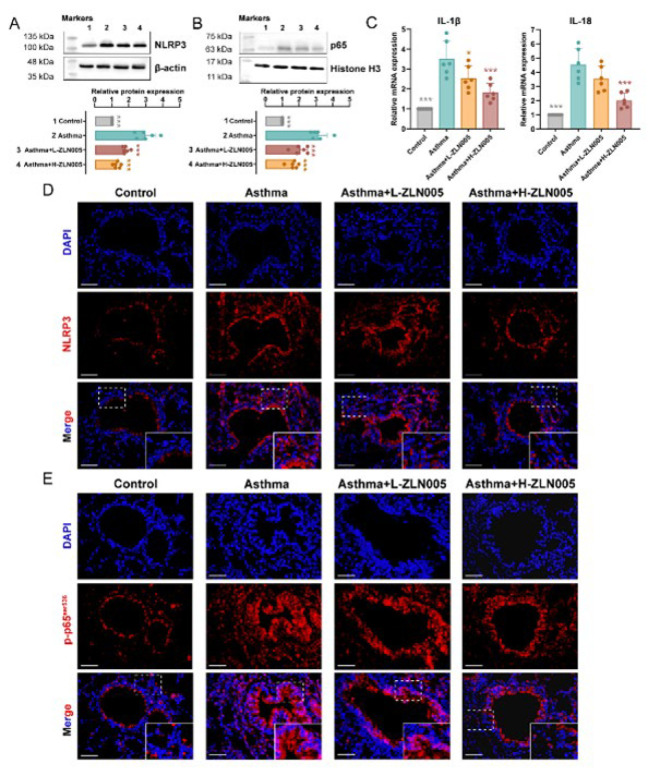
ZLN005 suppresses the NF-κB–p65/NLRP3 pathway activation in OVA-induced asthmatic mice

Western blot indicated that the levels of NLRP3 were significantly increased in the asthmatic mice compared to control mice. ZLN005 could down-regulate the levels of NLRP3 (Figure 4A). In addition, nuclear p65 was quantified by Histone H3 internal reference analysis, which showed that ZLN005 could restore the up-regulation of the p65 protein caused by OVA sensitization (Figure 4B). The mRNA levels of IL-1β and IL-18 were increased in OVA- sensitized asthmatic mice compared to control mice. At the same time, ZLN005 could alleviate this tendency (Figure 4C).

Additionally, immunofluorescence analysis indicated that the expression of NLRP3 in the control group was low. The expression of NLRP3 was elevated in the lung tissues in asthma mice. At the same time, ZLN005 alleviated the up-regulation of NLRP3 expression (Figure 4D). Similarly, immunofluorescence staining demonstrated the inhibitory effect of ZLN005 on p65 nuclear translocation in lung tissues of OVA-induced asthmatic mice (Figure 4E). These data are compelling evidence that ZLN005 regulates NLRP3-mediated lung injury proptosis by acting as an inhibitor of NF-κB activation during ZLN005 pathogenesis.

## Discussion

Asthma is one of the most prevalent chronic inflammatory diseases, affecting approximately 300 million people worldwide (31). It is estimated that up to 70% of childhood asthma cases may have an association with atopy or allergies (32). Herein, we established an OVA-sensitized asthma model in BALB/c mice. Our data revealed that the expression of PGC-1α is reduced in the lung tissues of asthmatic patients and mice. Notably, by suppressing IgE, OVA-specific IgE and inflammatory cytokine release, and inflammatory cell infiltration, the PGC-1α agonist (ZLN005) attenuated lung injury and inflammation. Mechanistically, ZLN005 resulted in a protective impact through the NF-κB/NLRP3 signaling pathway.

Allergic asthma is associated with increased production and enhanced total serum IgE levels (33). Reducing bronchial mucosal IgE levels, which can improve lung function in patients, is one of the therapeutic strategies for asthma (34). Previous studies have shown that inhaled terbutaline, a commercially available β2-agonist, effectively reduces plasma IgE and OVA-sIgE levels (35). Consistent with this research, our findings suggested that ZLN005 may reduce the expression of serum IgE and OVA-sIgE, thereby reducing inflammation in lung tissues.

Eosinophils, important cells in the progression of asthma and remodeling of the airways, can be activated by IL-5 and then migrate into the allergic airways (36). The activation of eosinophils releases pro-inflammatory and lipid mediators that can induce bronchoconstriction, mucus hypersecretion, and airway epithelial damage (37). Among the type 2 cytokines involved in initiating and maintaining eosinophilic airway inflammation, IL-3 and IL-5 are particularly important (38). Furthermore, asthma is typically associated with an exaggerated response by the immune system, which leads to the release of inflammatory mediators such as leukotrienes, interleukins, and cytokines. These mediators attract and activate inflammatory cells such as eosinophils, lymphocytes, and macrophages to infiltrate the peri bronchial tissue (39). Patients with symptomatic asthma have more peripheral neutrophils showing signs of activation (40). Our study demonstrated that ZLN005 attenuated OVA-induced up-regulation of IL-4, IL-5, and IL-13 in lung tissue and BALF of asthmatic mice. Meanwhile, ZLN005 reduced the number of mononuclear macrophages, lymphocytes, eosinophilic granulocytes, and neutrophils.

Exposure of animals to allergens, endotoxins, or microbial infections leads to increased activation of NF-κB (41, 42). In our study, NF-κB binding activity was significantly increased in OVA-sensitized mice. The 3-day treatment with ZLN005 (6 mg/kg; 12 mg/kg) markedly attenuated NF-κB activity in the lung tissues of OVA-sensitized mice. Furthermore, abnormal activation of the NF-κB signaling pathway could promote the immune response, cell proliferation/differentiation, and inflammation (43). Inhibition of NF-κB attenuates the expression of many cytokines, chemokines, and adhesion molecules involved in asthma pathology (44). Das *et al*. demonstrated that Th2 lymphocytes isolated from mice deficient in the p50 subunit of NF-κB produced significantly less IL-4, IL-5, and IL-13 than wild-type mice (45). Our study suggested that ZLN005 could regulate the expression level of Th2 cytokines through the NF-κB signaling pathway.

Accumulating clinical evidence has shed light on the role of NLRP3 inflammasome activation in the pathogenesis of asthma (46). Variants in the NLRP3 gene are associated with an increased risk of asthma in patients (47). In addition, expression of NLRP3 is higher in asthmatic BAL fluid than in healthy control BAL fluid (48). In our study, western blot and Immunofluorescence staining indicated that NLRP3 was up-regulated in lung tissues of asthmatic mice, while PGC-1a agonists (ZLN005) alleviated this tendency. The NLRP3 inflammasomes are well-characterized components of a protein complex that mediates the cleavage of pro-inflammatory cytokines pro-IL-1β and pro-IL-18 into their active, secreted forms (49). Furthermore, exaggerated activation of the NLRP3 inflammasome and production of IL-1β may be correlated with asthma disease severity (50, 51). Previous research has shown that the expression of the NLRP3 inflammasome and its downstream product, IL-1β, is increased in the BALF of a mouse model of asthma (52). Previous studies have shown that PGC-1α alleviates lung inflammation by regulating the expression of the inflammasome NLRP3 and down-regulating IL-1β (53). Our study showed that the ZLN005 reduced NLRP3 protein expression and IL-18 and IL-1β mRNA levels, suggesting that ZLN005 could alleviate asthma by inhibiting inflammasome activation.

(A) The content of PPARG Coactivator 1 Alpha (PGC-1α) in asthmatic patients. (B) Schematic diagram of animal experiment protocol. (C) mRNA expression of PGC-1α in lung tissues of asthmatic mice. (D) Representative western blot images and statistical analysis of PGC-1α in lung tissues asthmatic mice. (E) The expression levels of serum PGC-1α, immunoglobulin E (IgE), and OVA-specific IgE (OVA-sIgE). (F) Hematoxylin and eosin (H&E) stained sections from the lung tissues, scale bar= 100 μm. (G) Periodic acid-Schiff (PAS)-stained sections from the lung tissues, scale bar= 100 μm. *P<0.05, **P<0.01, ^###^*P*<0.001, ns: no significant difference.

(A) Experiment schematic of bronchoalveolar lavage fluid (BALF) collection and Diff-Quik staining. (B) Diff-Quik stained sections from BALF, scale bar= 50 μm. (C) The platelet counts of BALF. (D) The heatmap of inflammatory cells in BALF. *P<0.05, **P<0.01, ***P<0.001, ns: no significant difference.

(A) Content of interleukin (IL)-4 in BALF. (B) Content of IL-5 in BALF. (C) Content of IL-13 of BALF. (D) Content of IL-4 in lung tissues. (E) Content of IL-5 in lung tissues. (F) Content of IL-13 in lung tissues. *P<0.05, **P<0.01, ***P<0.001, ns: no significant difference.

(A) Representative western blot images and statistical analysis of NLRP3 in lung tissues. (B) Representative western blot images and statistical analysis of p65 in lung tissues. (C) mRNA expression levels of IL-1β and IL-18 in lung tissues. (D) Immunofluorescence staining of NLRP3 in lung tissues, scale bar= 50 μm. (E) Immunofluorescence staining of p-p65ser536 in lung tissues, scale bar= 50 μm. *P<0.05, **P<0.01, ***P<0.001, ns: no significant difference.

**Figure 5:**
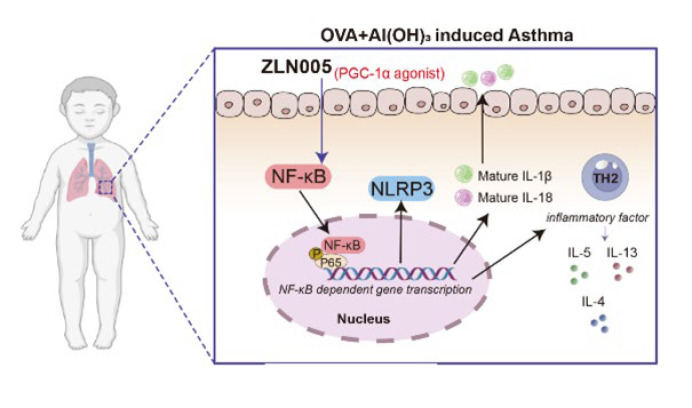
PGC-1a agonist (ZLN005) alleviates lung inflammation by OVA-induced asthma models through the NLRP3/P65 pathway

## Conclusion

In summary, our data demonstrate that ZLN005 modulates OVA-induced asthma exacerbations. PGC-1α agonist regulated lung inflammation and the release of inflammatory cytokines IL-4, IL-5, and IL-13 in asthmatic mice by inhibiting the NF-κB-p65/NLRP3 signaling pathway. Our findings provide insight into the underlying mechanisms and treatment targets in OVA-induced asthma exacerbations.

## Data Availability

All relevant data generated or analyzed during this study are included in this article. Further inquiries can be directed to the corresponding author.

## Declaration

There is no financial affiliation between any of the writers and any business that has an interest in the topic of this work.
